# CBX4 Drives Gastric Cancer Progression by Activating **β**-Catenin Signaling

**DOI:** 10.32604/or.2025.068651

**Published:** 2025-12-30

**Authors:** Wendong Jia, Ting Zhang, Ziying Zhang, Lingzhi Wu, Xihao Fu, Zhenxin Wang, Ni Yin

**Affiliations:** Department of Oncology, The First Affiliated Hospital of Soochow University, Suzhou, 215006, China

**Keywords:** Gastric cancer (GC), chromobox 4 (CBX4), proliferation, metastasis, β-catenin

## Abstract

**Objectives:**

Chromobox 4 (CBX4), a polycomb protein family member linked to tumor pathogenesis via dysregulation, has an incompletely defined role in gastric cancer (GC). The study aimed to investigate the role and mechanism of CBX4 in GC progression and evaluate its potential as a therapeutic target.

**Methods:**

CBX4 expression was assessed in GC tissues vs. adjacent non-cancerous tissues and in GC cell lines vs. normal gastric mucosal epithelial cells. Clinicopathological correlations were analyzed. Functional impacts of CBX4 were determined using knockdown and overexpression models *in vitro* (cell proliferation, migration, invasion) and *in vivo* (xenograft tumorigenesis in nude mice). Mechanistic studies evaluated β-catenin levels (total and nuclear) and transcriptional activity following CBX4 modulation. The functional dependency on Wnt/β-catenin signaling was tested using the pharmacological inhibitor XAV939 in CBX4-overexpressing cells.

**Results:**

CBX4 expression was significantly upregulated in GC tissues and cell lines. Elevated CBX4 levels strongly correlated with aggressive tumor characteristics, including larger tumor size, lymph node metastasis, and advanced Tumor, Node, Metastasis (TNM) stage. Functionally, CBX4 knockdown suppressed GC cell proliferation, migration, invasion *in vitro*, and tumorigenesis *in vivo*. Conversely, CBX4 overexpression enhanced these malignant traits. Mechanistically, CBX4 depletion reduced total and nuclear β-catenin levels and inhibited its transcriptional activity, while CBX4 overexpression had the opposite effect. Critically, XAV939-mediated inhibition of Wnt/β-catenin signaling attenuated the oncogenic effects induced by CBX4 overexpression.

**Conclusion:**

CBX4 upregulation promotes GC progression via β-catenin signaling activation. The CBX4/β-catenin axis emerges as a promising therapeutic target, offering potential for the development of precision treatment strategies in GC management.

## 1 Introduction

As the fifth most frequently diagnosed cancer globally, gastric cancer (GC) represents a significant contributor to the global disease burden, ranking as the fifth leading cause of cancer-related mortality [1]. Notably, China faces an alarmingly disproportionate share of this burden, accounting for nearly half of all newly diagnosed cases and associated deaths worldwide [2]. According to the World Health Organization (WHO) data (2020), GC is associated with 479,000 new cases and 374,000 deaths annually, positioning it as the fourth most prevalent malignancy in the nation and the third leading cause of cancer-related mortality [3]. Despite recent therapeutic advancements, including the integration of surgery with adjuvant therapies such as chemotherapy, the prognosis for GC patients remains unsatisfactory [4,5]. Consequently, there is an urgent need to delve deeper into the molecular mechanisms underlying the onset and malignant progression of GC. Such insights are crucial for identifying promising biomarkers for early diagnosis and validating critical therapeutic targets to improve intervention strategies and enhance patient outcomes.

The chromobox (CBX) protein family consists of eight members (CBX1-8) in mammalian cells. Based on their molecular framework, CBX proteins are categorized into two classes: 1) the heterochromatin protein 1 (HP1) class, which includes CBX1, CBX3, and CBX5; and 2) the polycomb group (PcG) class, a critical component of the polycomb-repressive complex 1 (PRC1), encompassing CBX2, CBX4, CBX6, CBX7, and CBX8 [6]. The HP1 and PcG classes are involved in epigenetic regulation and gene transcriptional repression. They achieve this through their chromodomains, which recognize histone H3 trimethylation at lysine 9 (H3K9me3) and H3K27me3, respectively [6]. CBX4, a transcriptional repressor within the PcG class of CBX proteins, participates in PRC1 recruitment to specific chromatin regions via its N-terminal chromodomain [6]. Additionally, CBX4 possesses two unique sumoylation (SUMO)-interacting motifs (SIMs), conferring it SUMO E3 ligase activity [7,8]. For instance, CBX4 can suppress the transcription of fibroblast growth factor receptor 3 (FGFR3), cyclin A, and p16INK4a by binding to their gene promoters [9–11]. As a SUMO E3 ligase, CBX4 has multiple identified substrates, such as Smad-interacting protein 1 (SIP1) [12], DNA methyltransferase 3a (Dnmt3a) [13], B-cell specific moloney murine leukemia virus insertion site 1 (BMI1) [14], C-terminal binding protein (CtBP)-interacting protein (CtIP) [15], hypoxia-inducible factor 1α (HIF1α) [16], and PR domain-containing 16 (Prdm16) [17]. Thus, CBX4 plays a significant role in regulating cellular activities through both PRC1-dependent and -independent mechanisms.

Emerging evidence indicates that CBX4 dysregulation plays a crucial role in carcinogenesis and tumor progression. In hepatocellular carcinoma (HCC), CBX4 expression is significantly upregulated, with its levels positively correlated with tumor malignancy and poor patient prognosis [18]. Importantly, CBX4 serves as an independent prognostic factor for HCC patients undergoing postoperative transarterial chemoembolization (TACE) [19]. Mechanistically, CBX4 enhances the transcriptional activity of HIF1α through SUMO modification, thereby promoting HCC progression [20]. In breast cancer, CBX4 is overexpressed and activates oncogenic Notch1 signaling by transcriptionally repressing miR-137 [21]. Additionally, in lung cancer, CBX4 drives tumor growth and metastasis by upregulating BMI1 [22] and stimulating the Wnt/β-catenin signaling pathway [23]. In osteosarcoma, CBX4 has been shown to enhance tumorigenesis by activating HIF1α signaling [24] and by recruiting the histone acetyltransferase general control nonderepressible 5 (GCN5) to upregulate runt-related transcription factor 2 (Runx2) [25]. In renal carcinoma, CBX4 interacts with histone deacetylase 1 (HDAC1) to suppress the transcriptional activity of the Kruppel-like factor 6 (KLF6) promoter, leading to reduced KLF6 expression and tumor-promoting effects [26]. However, in colorectal cancer, CBX4 exhibits tumor-suppressive effects by recruiting HDAC3 to maintain the deacetylation of histone H3K27, thereby repressing Runx2 expression [27]. Collectively, these findings demonstrate that CBX4 can either promote or inhibit tumor progression depending on the cell type, its interacting molecules, and the downstream target genes involved.

Luo et al. [28] examined the link between the CBX4 rs77447679 polymorphism and GC pathogenesis, demonstrating a strong association with GC susceptibility. Bioinformatics analysis by Lin et al. [29] revealed increased CBX4 expression in GC, correlating with clinicopathological features like tumor stage/grade, lymph node metastasis, and *Helicobacter pylori* infection, as well as with patient prognosis. While evidence points to CBX4 acting as an oncogenic driver in GC, its precise role requires further exploration. Consequently, tissues from GC patients were collected to quantify CBX4 expression and analyze its clinicopathological correlations. Transgenic GC cell lines with CBX4 overexpression and knockdown were then established. Using *in vitro* models and *in vivo* nude mouse xenografts, the study aimed to clarify CBX4’s roles in GC cell propagation, development, and metastasis, along with the potential molecular pathways.

## 2 Materials and Methods

### 2.1 Molecular and Cellular Biology Reagents

RPMI-1640 medium (L210KJ) and phosphate-buffered saline (PBS) (B310KJ) were supplied by BasalMedia (Shanghai, China). Fetal bovine serum (FBS) (C04001) was supplied by VivaCell (Shanghai, China). Penicillin-streptomycin (C0222), trypsin-EDTA solution (C0223), western blot (WB) lysis buffer (P0013B), nuclear extraction kit (P0028), phenylmethanesulfonyl fluoride (ST507), bicinchoninic acid protein assay kit (P0010), the BeyoECL Star chemiluminescence detection kit (P0018AS), Cell counting kit-8 (CCK-8) (C0038), puromycin (ST551), and blasticidin S (ST018) were supplied by Beyotime Biotechnology (Shanghai, China). 10% neutral formalin (G2161), 4% paraformaldehyde (P1110), 0.5% crystal violet (G1065), and diaminobenzidine (DAB) (DA1010) were supplied by Solarbio (Beijing, China). Matrigel (356234) was supplied by Corning (Corning, NY, USA). Lipofectamine 2000 (11668019) was supplied by Thermo Fisher Scientific (Waltham, MA, USA). XAV939 (HY-15147) was supplied by MCE (Monmouth Junction, NJ, USA). The dual-luciferase (LUC) assay system (E2920) was provided by Promega (Madison, WI, USA).

### 2.2 Antibodies (Ab)

Rabbit anti-CBX4 (WB 1:1000, IHC 1:200, GTX53928) was purchased from GeneTex (Irvine, CA, USA). Rabbit anti-β-catenin (WB 1:1000, #8480) and rabbit anti-Histone H3 (WB 1:1000, #9717) were supplied by CST (Danvers, MA, USA). Rabbit anti-GAPDH (WB 1:3000, YM8394) was purchased from ImmunoWay (Plano, TX, USA). Horseradish peroxidase (HRP)-conjugated goat anti-rabbit IgG (WB 1:5000, IHC 1:200, G1213) was obtained from Servicebio (Wuhan, China).

## 3 Lentiviruses and LUC Reporter Plasmids

The lentiviral vectors were constructed as follows: LV-shCBX4 (encoding human CBX4 shRNA, puromycin N-acetyltransferase, and GFP) and the control LV-shNC (negative control shRNA) were obtained from Hanbio (Shanghai, China); LV-CBX4 (expressing human CBX4, blasticidin S deaminase, and GFP) and the blank control LV were obtained from Novobio (Shanghai, China). The reporter plasmids TCF/LEF1-LUC (firefly) and pGMLR-TK-LUC (Renilla) were purchased from Genomeditech (Shanghai, China).

### 3.1 Cell Culture and Generation of Transgenic Cell Lines

The human GC cell lines AGS and HGC-27, along with the gastric mucosal epithelial cell line GES-1, were obtained from the Cell Bank of the Chinese Academy of Sciences (Shanghai, China). Additional human GC cell lines (MKN45, N87, SNU-1) were acquired from Procell (Wuhan, Hubei, China). All parental cell lines were cultured in complete RPMI-1640 medium supplemented with 10% FBS and 1% penicillin-streptomycin under standard conditions (37°C, 5% CO_2_). Using lentiviral transduction methods [30,31], we established transgenic GC cell lines: MKN45-shCBX4 (CBX4 knockdown) and MKN45-shNC (negative control for CBX4 knockdown); SNU-1-CBX4 (CBX4 overexpression) and SNU-1-NC (negative control for CBX4 overexpression). The MKN45-shCBX4 and MKN45-shNC transgenic cell lines were maintained in culture medium containing 2 μg/mL puromycin, while the SNU-1-CBX4 and SNU-1-NC transgenic cell lines were cultured in medium supplemented with 10 μg/mL blasticidin S. All cell lines were authenticated using short tandem repeat profiling and tested negative for mycoplasma contamination prior to experiments.

### 3.2 GC Tissue Specimens and Tissue Microarray (TMA) Preparation

A total of 114 pairs of human GC tumor (T) and adjacent non-tumor (N) tissues were obtained from 114 patients who had undergone surgery but received no neoadjuvant therapy at the First Affiliated Hospital of Soochow University (Suzhou, China) from July 6, 2016, to November 24, 2017. Snap-frozen in liquid nitrogen and fixed in 10% neutral buffered formalin, the specimens were then stored at −80°C or processed for paraffin embedding. GC TMAs were constructed from the fixed specimens following established protocols [30]. The Ethics Committee of the First Affiliated Hospital of Soochow University approved the study (approval no. 2022064), and written informed consent was obtained from all patients.

### 3.3 Immunohistochemistry (IHC) Analysis

Paraffin sections underwent dewaxing through sequential immersion in environmentally friendly dewaxing solutions I-III (10 min each) (G1128, Servicebio), graded ethanols (100% I-III, 5 min each), and distilled water. Antigen retrieval was performed by microwave-heating in EDTA buffer (pH 9.0) (G1203, Servicebio), ensuring sections remained submerged to prevent drying. After natural cooling, slides were washed in PBS (pH 7.4; 3 × 5 min) with agitation. Endogenous peroxidase activity was blocked by incubating sections in 3% H_2_O_2_ at room temperature (RT) for 25 min in the dark, followed by PBS washes (3 × 5 min). Sections were blocked with 3% BSA (GC305010, Servicebio) at RT for 30 min. After gentle removal of the blocking solution, rabbit anti-CBX4 primary antibody (1:200; GTX53928) was applied, and slides were incubated overnight at 4°C in a humidified chamber. Following PBS washes (3 × 5 min), an HRP-conjugated goat anti-rabbit secondary antibody (1:200; G1213) was applied and incubated at RT for 50 min. After further PBS washes (3 × 5 min), freshly prepared DAB chromogen solution (DA1010, Solarbio) was applied. Development time was monitored microscopically (positive signal: brownish-yellow) and stopped by rinsing under tap water. Nuclei were counterstained with hematoxylin (3 min) (G1004, Servicebio), rinsed, differentiated briefly in hematoxylin differentiation solution (G1039, Servicebio), rinsed again, blued in hematoxylin bluing solution (G1040, Servicebio), and given a final tap water rinse. Sections were dehydrated through graded ethanols (75%, 85%, and absolute I & II; 5 min each), cleared in n-butanol (5 min) and xylene I (5 min), air-dried briefly, and mounted with coverslips using mounting medium. Staining was evaluated using brightfield microscopy (E100, Nikon, Shanghai, China). A weighted IHC scoring system was employed, with high CBX4 expression defined as a score ≥4 (++/+++) and low expression as <4 (−/+) [30].

### 3.4 CCK-8 Assay

Cell proliferation was assessed using the CCK-8 assay. MKN45-shCBX4 and MKN45-shNC cells, as well as SNU-1-CBX4 and SNU-1-NC cells, were seeded in 96-well plates at a density of 1 × 10^4^ cells per well with 200 μL of culture medium. On the 1st, 2nd, 3rd, and 4th day following cell culture, 20 μL of CCK-8 reagent (C0038, Beyotime Biotechnology, Shanghai, China) was added to the cell culture medium. Cell proliferation was monitored daily over four consecutive days, and absorbance values at 450 nm were measured each day using a microplate reader (Synergy H1, BioTek, Winooski, VT, USA) according to the manufacturer’s instructions.

### 3.5 Plate Colony Formation Assay

Clonogenic ability was evaluated using the plate colony formation assay. MKN45-shCBX4 and MKN45-shNC cells, as well as SNU-1-CBX4 and SNU-1-NC cells, were seeded in 6-well plates at 200 cells per well with 2 mL of culture medium. After a 14-day culture period, colonies were fixed with 4% paraformaldehyde and stained with 0.5% crystal violet. The number of colonies was quantified using ImageJ software (version 1.8, National Institutes of Health, Bethesda, MD, USA) to assess clonogenic capacity.

### 3.6 Transwell-Based Migration and Invasion Assays

Cell migration and invasion were assessed using Transwell assays with 8-μm pore size 24-well chambers [29,30]. For migration assays, cells (MKN45-shCBX4 vs. MKN45-shNC; SNU-1-CBX4 vs. SNU-1-NC) were seeded in serum-free medium at 2 × 10^4^ cells/200 μL in uncoated chambers. For invasion assays, cells were seeded at 2 × 10^5^ cells/200 μL in Matrigel-coated chambers. After 24 h of culture, migrated or invaded cells were fixed with 4% paraformaldehyde and stained with 0.5% crystal violet. The brightfield microscope was employed for image capturing, whereas ImageJ software (version 1.8, National Institutes of Health) was adopted for counting to evaluate migration and invasion capacities.

### 3.7 Subcutaneous Xenograft Assay in Nude Mice

Experiments utilized 12 four-week-old female athymic BALB/c nude mice obtained from Shanghai SLAC Laboratory Animal Co., Ltd. (Laboratory Animal Production License: SCXK (Shanghai) 2022-0004, Shanghai, China). All the animals were housed in individually ventilated cages under specific pathogen-free (SPF) conditions at 22 ± 1°C, 50 ± 1% humidity, and a 12:12-h light/dark cycle, with free access to autoclaved food and water. After a two-week acclimatization period in the animal facility of Soochow University (Laboratory Animal Use License: SCXK (Jiangsu) 2022-0008, Suzhou, China), nude mice were randomly assigned to two groups and received subcutaneous injections of MKN45-shCBX4 or MKN45-shNC cells (2 × 10^6^ cells per mouse, five mice per group) to establish GC xenografts. Tumor growth was monitored using volumetric assessment (*V* = $ab^{2}/2$, where ‘α’ and ‘b’ represent the longest and shortest diameters, respectively). Mice were euthanized by cervical dislocation at four weeks post-inoculation, followed by tumor resection and gravimetric analysis. All animal procedures complied with the regulations and guidelines of Soochow University institutional animal care and followed the Association for Assessment and Accreditation of Laboratory Animal Care (AAALAC)/Institutional Animal Care and Use Committee (IACUC) guidelines. This study received ethical approval from Soochow University’s Animal Research Ethics Committee (approval no. SUDA20220526A01).

### 3.8 Fluorescence Microscopic Analysis

GC cells, including MKN45-shCBX4, MKN45-shNC, and MKN45 (negative control), as well as SNU-1-CBX4, SNU-1-NC, and SNU-1 (negative control), were cultured for 48 h. The cells were then observed under fluorescence microscopy (NIB600, Nexcope, Ningbo, China) at the same field of vision using bright field (BF) and GFP fluorescence field, respectively.

### 3.9 Flow Cytometric Analysis

GC cells, including MKN45-shCBX4, MKN45-shNC, and MKN45 (negative control), as well as SNU-1-CBX4, SNU-1-NC, and SNU-1 (negative control), were resuspended in PBS (1×, pH 7.4) at a density of 1 × 10^6^ cells/mL. Cellular GFP expression was quantified by flow cytometry (FACSCelesta™, BD, Franklin Lakes, NJ, USA) using excitation at 488 nm and emission at 510 nm.

## 4 WB Analysis

Whole-cell or nuclear lysates from snap-frozen GC T/N tissues and parental/transgenic cells (MKN45-shCBX4, MKN45-shNC, SNU-1-CBX4, SNU-1-NC) underwent WB analysis for CBX4, β-catenin, GAPDH, and Histone H3 as previously described [30]. The anti-CBX4 (1:1000), anti-β-catenin (1:1000), anti-GAPDH (1:3000), or anti-Histone H3 (1:1000) primary antibody and HRP-conjugated goat anti-rabbit IgG (1:5000) secondary antibody were applied according to the manufacturer’s protocols. The protein signaling on the membranes was detected using a BeyoECL Star chemiluminescence detection kit (P0018AS, Beyotime Biotechnology, Shanghai, China), and the protein bands were then visualized after their exposure to a fully automated chemiluminescence image analyzer (Tanon 5200, Shanghai, China). Band densities were quantified and normalized to GAPDH or Histone H3 loading control.

### 4.1 Dual Luciferase Reporter Gene Assay

MKN45-shCBX4 and MKN45-shNC cells, as well as SNU-1-CBX4 and SNU-1-NC cells, were cultured in 96-well plates at a density of 1 × 10^4^ cells per well with 200 μL of culture medium for 24 h and co-transfected with TCF/LEF1-LUC and pGMLR-TK-LUC reporter plasmids at a ratio of 50:1 using Lipofectamine 2000. After 72 h of transfection, cells were lysed with passive lysis buffer (20 μL/well), and firefly and Renilla LUC activities were detected using LUC Reagent II (100 μL/well) and Stop & Glo Reagent (100 μL/well), respectively, following the manufacturer’s instructions. The relative firefly LUC activity (β-catenin transcriptional activity) of the cells was calculated.

### 4.2 Wnt/***β***-Catenin Inhibition Assays

SNU-1-CBX4 GC cells were treated with XAV939, a Wnt/β-catenin inhibitor, at a concentration of 10 μM or with DMSO (vehicle control), followed by Wnt/β-catenin inhibition functional assays. Commonly cultured SNU-1-CBX4 and SNU-1-NC GC cells were also included in the assays.

### 4.3 Bioinformatics Analysis

We used the University of Alabama at Birmingham cancer data analysis (UALCAN, https://ualcan.path.uab.edu/index.html) (accessed on 26 August 2025) [32] and gene expression profiling interactive analysis (GEPIA2.0, http://gepia2.cancer-pku.cn/#index) (accessed on 26 August 2025) databases to comprehensively analyze CBX4 expression data. We then used the Kaplan-Meier plotter (https://www.kmplot.com/analysis/) (accessed on 26 August 2025) database [33] to comprehensively analyze CBX4 prognosis data. Briefly, the results from public databases were obtained using automated statistical analysis.

### 4.4 Statistical Analysis

Immunohistochemical staining results were evaluated using a four-tier scoring scale (−, +, ++, +++). The proportions of cohorts with high and low expression levels were calculated. For continuous variables, normal distribution was assessed; parametric data (with *p* > 0.1 for normality) are expressed as mean±standard deviation. After confirming variance homogeneity (via Levene’s test, *p* > 0.1), one-way ANOVA with Least Significant Difference (LSD) post-hoc tests was performed. Statistical analyses were conducted using SPSS 20.0 (IBM Corp., Armonk, NY, USA) and included the Mann-Whitney U test, Pearson’s χ^2^ test, Log-rank test, Student’s *t*-test, and ANOVA. A two-tailed *p*-value of < 0.05 was considered statistically significant.

## 5 Results

### 5.1 CBX4 Overexpression in GC Tissues Correlates with Clinicopathological Parameters and Poor Prognosis

**Figure 1:**
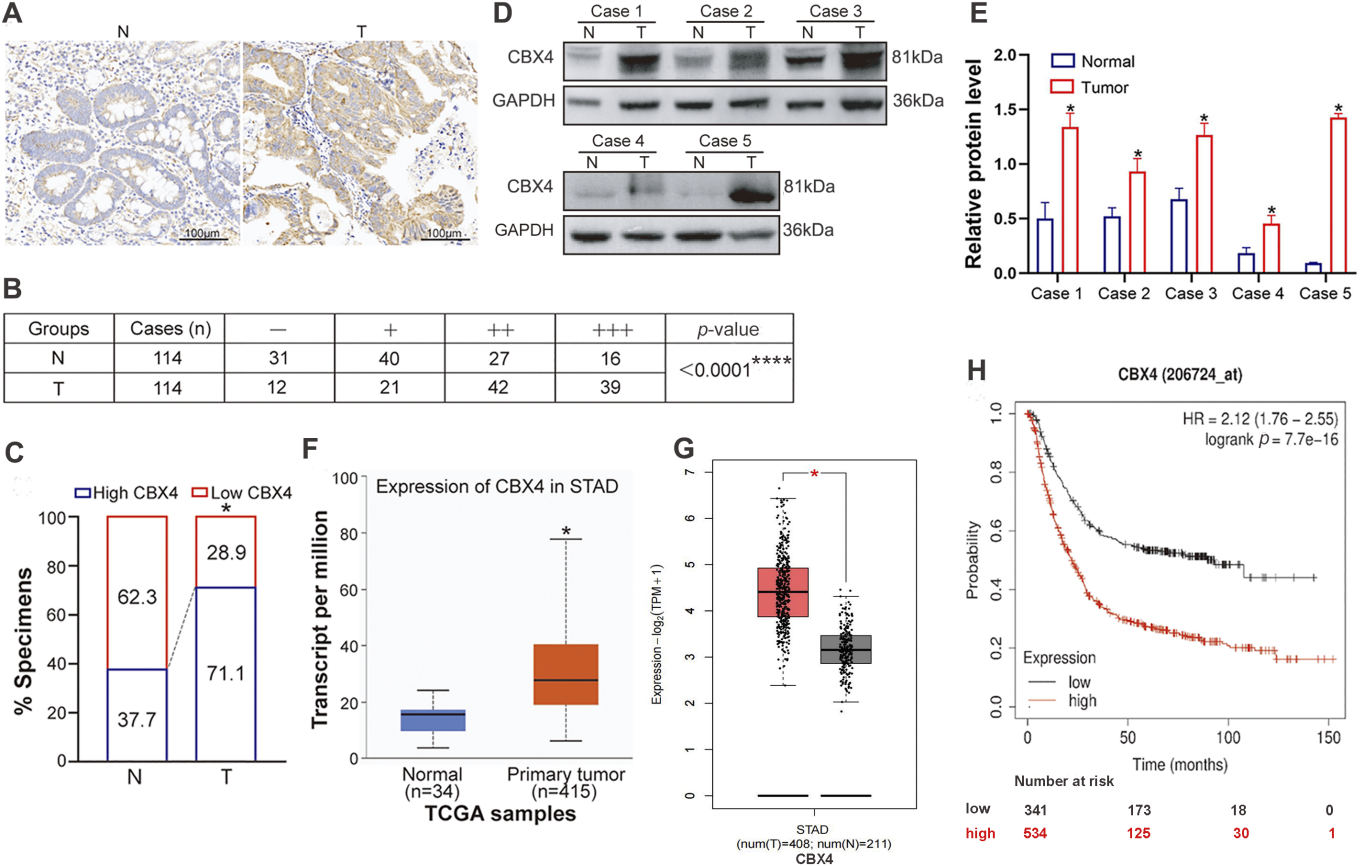
CBX4 expression is upregulated in GC tissues, and its elevation is associated with clinical malignant characteristics and poor survival of GC patients. (**A**) Representative IHC images of CBX4 in GC tumor (T) tissues and matched normal (N) tissues. (**B**) IHC scoring analysis of CBX4 in 114 paired GC tumor and normal tissues, showing significantly higher expression in tumor tissues. ****p < 0.0001, Mann-Whitney U test. High CBX4 expression is defined as a score ≥4 (++/+++) and low expression as <4 (−/+). (**C**) Proportion of high vs. low CBX4 expression in GC tumor and matched normal tissues, revealing a markedly higher prevalence of high CBX4 expression in tumor samples. *p < 0.05, Pearson’s χ^2^ test. (**D**) Representative Western blot analysis of CBX4 protein levels in GC tumor and matched normal tissues. (**E**) Relative protein levels of CBX4 in paired GC normal and tumor tissues (Cases 1–5). *p < 0.05, Student’s *t*-test. (**F**) mRNA expression analysis of CBX4 in GC tumor tissues vs. normal gastric tissues using the UALCAN database, demonstrating elevated CBX4 mRNA levels in tumor tissues. *p < 0.05, Student’s *t*-test. (**G**) Validation of CBX4 mRNA overexpression in GC tumor tissues compared to normal gastric tissues via the GEPIA database. *p < 0.05, Student’s *t*-test. (**H**) Kaplan-Meier survival analysis indicating that high CBX4 expression correlates with poorer overall survival in GC patients. *p* = 7.7e−16, Log-rank test

Immunohistochemistry (IHC) analysis of a human GC tissue microarray (TMA) containing 114 paired tumor tissues (T) and adjacent non-tumor tissues (N) (Fig. 1A) revealed that 71.1% of tumor samples exhibited high CBX4 expression (39 cases ‘+++’ and 42 cases ‘++’), while 28.9% had low expression (21 cases ‘+’ and 12 cases ‘−’) (Fig. 1B,C). In contrast, only 37.7% of adjacent normal tissues showed high CBX4 expression (16 cases ‘+++’ and 27 cases ‘++’), whereas 62.3% had low CBX4 levels (40 cases ‘+’ and 31 cases ‘−’) (Fig. 1B,C). GC T tissues showed significantly higher CBX4 protein levels than adjacent N tissues (*p* < 0.05). Western blot (WB) analysis also confirmed tumor-specific CBX4 upregulation in paired T/N samples (*p* < 0.05; Fig. 1D,E). TCGA data analysis via UALCAN (Fig. 1F) and GEPIA (Fig. 1G) similarly indicated elevated CBX4 mRNA levels in GC (*p* < 0.05), consistent with prior studies [29]. Collectively, these findings confirm CBX4 overexpression in human GC tissues.

To assess the correlation between CBX4 expression levels in GC tissues and clinicopathological characteristics, among the 114 GC patients in our study, 81 patients with CBX4 IHC scores of “++” and “+++” in GC tissues were categorized into the CBX4 high expression group, while 33 patients with CBX4 IHC scores of “−” and “+” were classified into the CBX4 low expression group. The analysis of clinicopathological variables demonstrated that high CBX4 expression was significantly associated (*p* < 0.05) with larger tumor size, lymph node metastasis, and advanced TNM stage (Table 1). Kaplan-Meier survival analysis of data from 875 GC patients, categorized by CBX4 mRNA levels, confirmed that elevated CBX4 expression was correlated with substantially reduced overall survival (*p* < 0.05) (Fig. 1H). These findings, supported by TCGA data, indicate that CBX4 upregulation serves as a marker associated with malignant clinicopathological features and unfavorable prognosis in GC, suggesting its potential role as a driver of tumor aggressiveness.

**Table 1:** Correlation between CBX4 expression levels and clinicopathological parameters in GC patients

Variables	Low CBX4 (n = 33)	High CBX4 (n = 81)	*p*-value
Age (years)			
<60	8	17	0.703
>60	25	64	
Sex			
Male	20	56	0.381
Female	13	25	
Tumor differentiation			
Well/moderately differentiated	9	24	0.801
Poorly differentiated	24	57	
Tumor diameter (cm)			
<5	24	36	0.006*
>5	9	45	
Lymph node metastasis			
Negative	12	12	0.010*
Positive	21	69	
TNM stage			
I+II	14	16	0.013*
III+IV	19	65	

Note: *p < 0.05, Pearson’s χ^2^ test.

### 5.2 CBX4 Upregulation and Knockdown/Overexpression Models in GC Cell Lines

**Figure 2:**
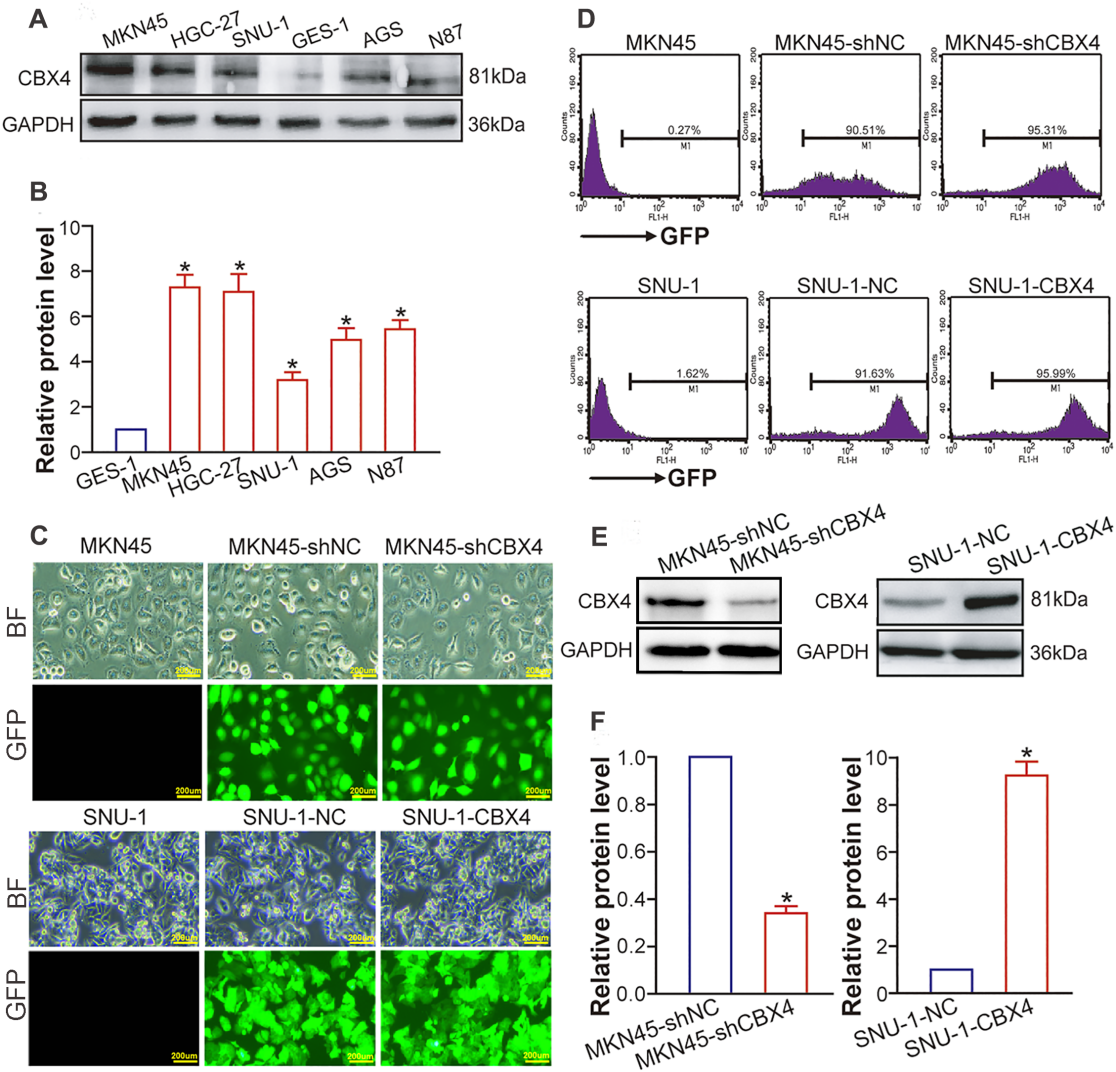
CBX4 is increased in GC cells and lentivirus-directed CBX4 knockdown or overexpression. (**A**) Representative Western blot analysis of CBX4 protein levels in GC cell lines and the gastric mucosal epithelial cell line. (**B**) Relative protein levels of CBX4 in GC cell lines, normalized to GES-1 gastric mucosal epithelial cells (set to 1). *p < 0.05 vs. GES-1 control, ANOVA with LSD post hoc multiple comparisons. (**C**) Fluorescence microscopic imaging of GFP expression in transgenic and parental GC cell lines. MKN45 served as a negative control for MKN45-shCBX4 and MKN45-shNC; SNU-1 served as a negative control for SNU-1-CBX4 and SNU-1-NC. (**D**) Flow cytometric analysis of GFP expression in transgenic and parental GC cell lines. MKN45 was used as a negative control for MKN45-shCBX4 and MKN45-shNC; SNU-1 was used as a negative control for SNU-1-CBX4 and SNU-1-NC. (**E**) Western blot analysis of CBX4 protein levels in transgenic GC cell lines after lentivirus-mediated CBX4 knockdown or overexpression. (**F**) Quantification of relative CBX4 protein levels in CBX4-knockdown (MKN45-shCBX4 vs. MKN45-shNC) and CBX4-overexpressing (SNU-1-CBX4 vs. SNU-1-NC) GC cell lines. *p < 0.05, Student’s *t*-test

Our WB analysis demonstrated significantly elevated CBX4 protein levels in GC parental cell lines relative to the normal gastric mucosal epithelial cell line GES-1 (*p* < 0.05; Fig. 2A and B), aligning with the CBX4 upregulation documented in clinical GC tissues. For functional studies, we established transgenic GC cell lines: MKN45 cells were transduced with lentiviral vectors expressing CBX4 shRNA to suppress expression, while SNU-1 cells were infected with CBX4-overexpression constructs to enhance protein levels. Successful transduction was confirmed via GFP fluorescence microscopy and flow cytometry (Fig. 2C and D). The efficiency of CBX4 knockdown and overexpression was validated by WB (Fig. 2E and F). Collectively, these data confirm the successful establishment of CBX4-knockdown MKN45 cells and CBX4-overexpressing SNU-1 cells for functional investigations.

### 5.3 CBX4 Promotes GC Cell Proliferation In Vitro and Tumorigenicity In Vivo

**Figure 3:**
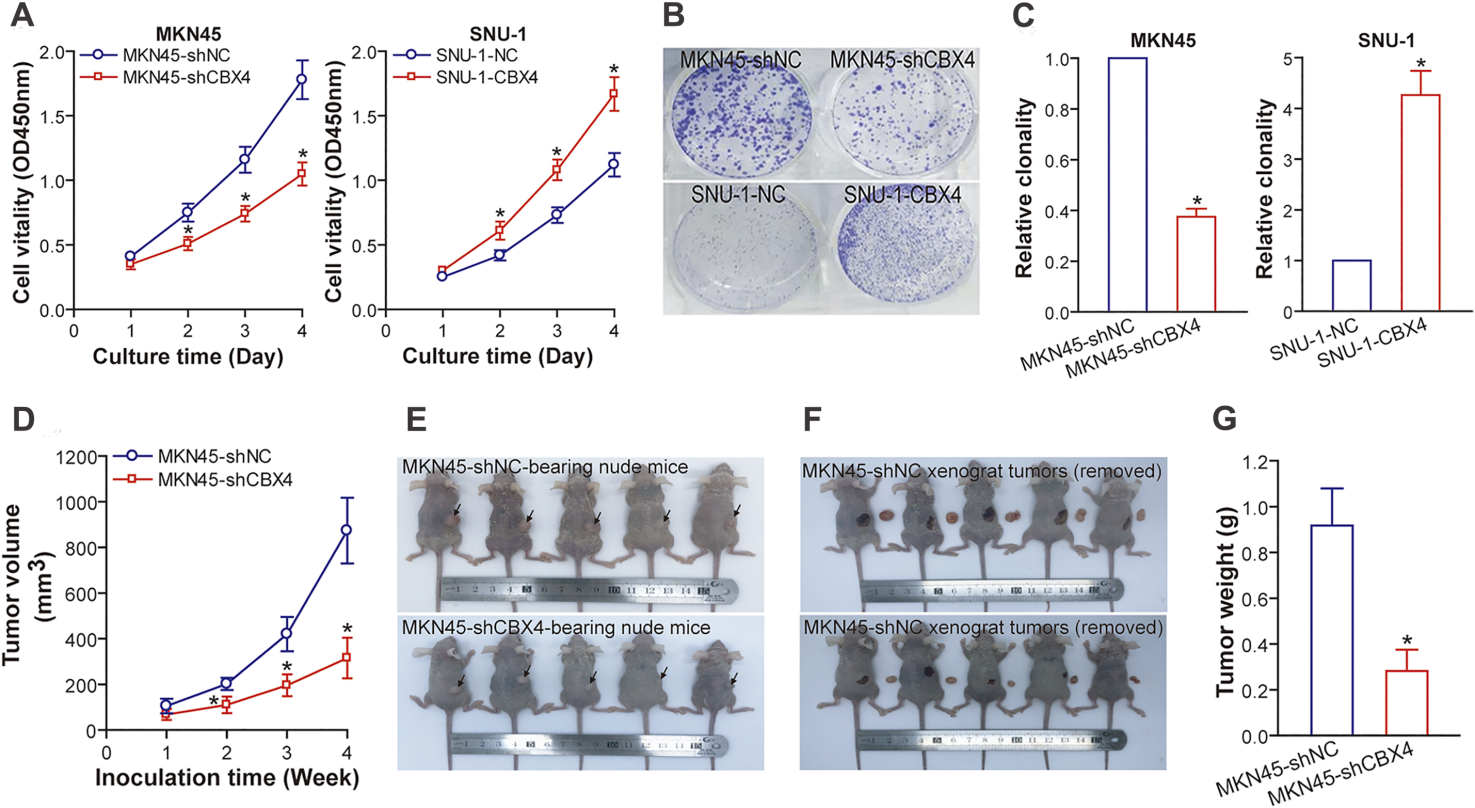
CBX4 accelerates GC cell proliferation *in vitro* and promotes the growth of GC xenograft tumors in nude mice. (**A**) CCK-8 assay results indicate that CBX4 depletion in MKN45-shCBX4 cells significantly reduced cell viability compared to MKN45-shNC controls (*p < 0.05 at days 2, 3, and 4 post-culture), whereas CBX4 overexpression in SNU-1-CBX4 cells enhanced cell viability relative to SNU-1-NC controls (*p < 0.05 at days 2, 3, and 4 post-culture), as assessed by Student’s *t*-test. (**B**) Representative images of colony formation assays in CBX4-modulated GC cell lines. (**C**) Quantification of clonogenic potential in CBX4-knockdown (MKN45-shCBX4 vs. MKN45-shNC) and CBX4-overexpressing (SNU-1-CBX4 vs. SNU-1-NC) GC cells. Values are normalized to respective controls (set to 1). *p < 0.05, Student’s *t*-test. (**D**) Tumor volume measurements in nude mice xenografted with GC cells. CBX4 knockdown markedly reduced tumor volume compared to MKN45-shNC controls at weeks 2, 3, and 4 post-inoculation (*p < 0.05, Student’s *t*-test). (**E**) Images of nude mice bearing GC xenografts. (**F**) Gross appearance of GC xenograft tumors excised from nude mice. (**G**) Tumor weight analysis of excised xenografts. *p < 0.05, Student’s *t*-test

Employing our genetically engineered GC cell models, we delineated the functional ramifications of CBX4 modulation. *In vitro* experiments revealed that CBX4 knockdown markedly suppressed MKN45 cell proliferation, as quantified by CCK-8 assay (Fig. 3A), and markedly reduced clonogenic potential, as demonstrated by plate colony formation assay (Fig. 3B and C), relative to shNC counterparts (*p* < 0.05). Reciprocally, CBX4 overexpression bolstered these proliferative attributes in SNU-1 cells when compared to NC cells (*p* < 0.05; Fig. 3A–C). *In vivo*, nude mice inoculated with MKN45-shCBX4 cells developed subcutaneous xenografts with significantly attenuated tumor growth, evidenced by reduced volume and weight (*p* < 0.05), compared to those from MKN45-shNC cells (Figs. 3D–3G). Collectively, these functional perturbation studies position CBX4 as a pivotal driver of GC cell proliferation and tumorigenesis.

### 5.4 CBX4 Drives GC Cell Migration and Invasion

**Figure 4:**
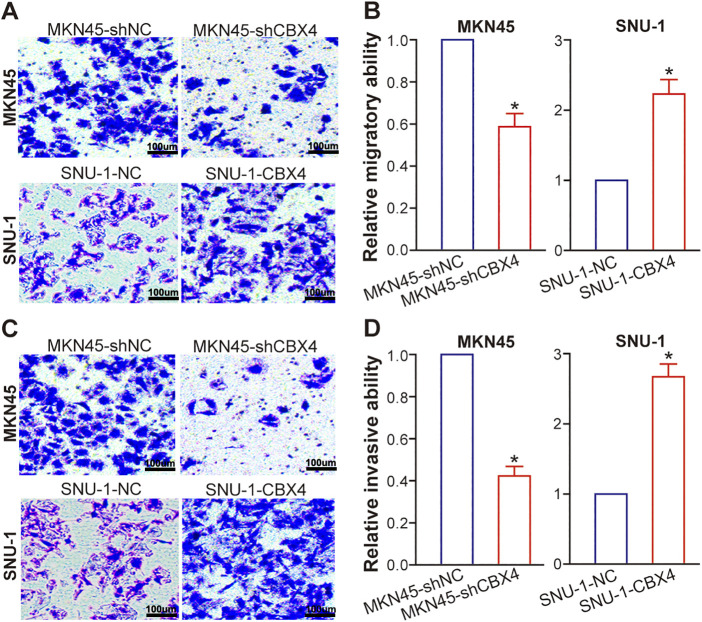
CBX4 enhances GC cell migration and invasion. (**A**) Representative images of transwell migration assays in CBX4-modulated GC cell lines. (**B**) Quantification of migratory capacity in CBX4-knockdown (MKN45-shCBX4 vs. MKN45-shNC) and CBX4-overexpressing (SNU-1-CBX4 vs. SNU-1-NC) GC cells. Values are normalized to respective controls (set to 1). *p < 0.05, Student’s *t*-test. (**C**) Representative images of transwell invasion assays in CBX4-modulated GC cell lines. (**D**) Quantification of invasive capacity in CBX4-knockdown and CBX4-overexpressing GC cells. MKN45-shNC served as a control for MKN45-shCBX4 (set to 1), and SNU-1-NC served as a control for SNU-1-CBX4 (set to 1). *p < 0.05, Student’s *t*-test

Employing transwell assays, we evaluated the metastatic potential of GC cells following CBX4 modulation. In MKN45 cells, CBX4 knockdown markedly curtailed migration (Fig. 4A and B; *p* < 0.05) and invasion (Fig. 4C and D; *p* < 0.05) relative to MKN45-shNC control cells. In contrast, CBX4 overexpression in SNU-1 cells robustly bolstered both migratory and invasive capabilities when compared to SNU-1-NC control cells (Figs. 4A–D; *p* < 0.05). Collectively, these findings demonstrate that CBX4 plays a driving role in augmenting the metastatic potential of GC cells.

### 5.5 CBX4 Promotes GC Progression via **β**-Catenin Signaling Activation

**Figure 5:**
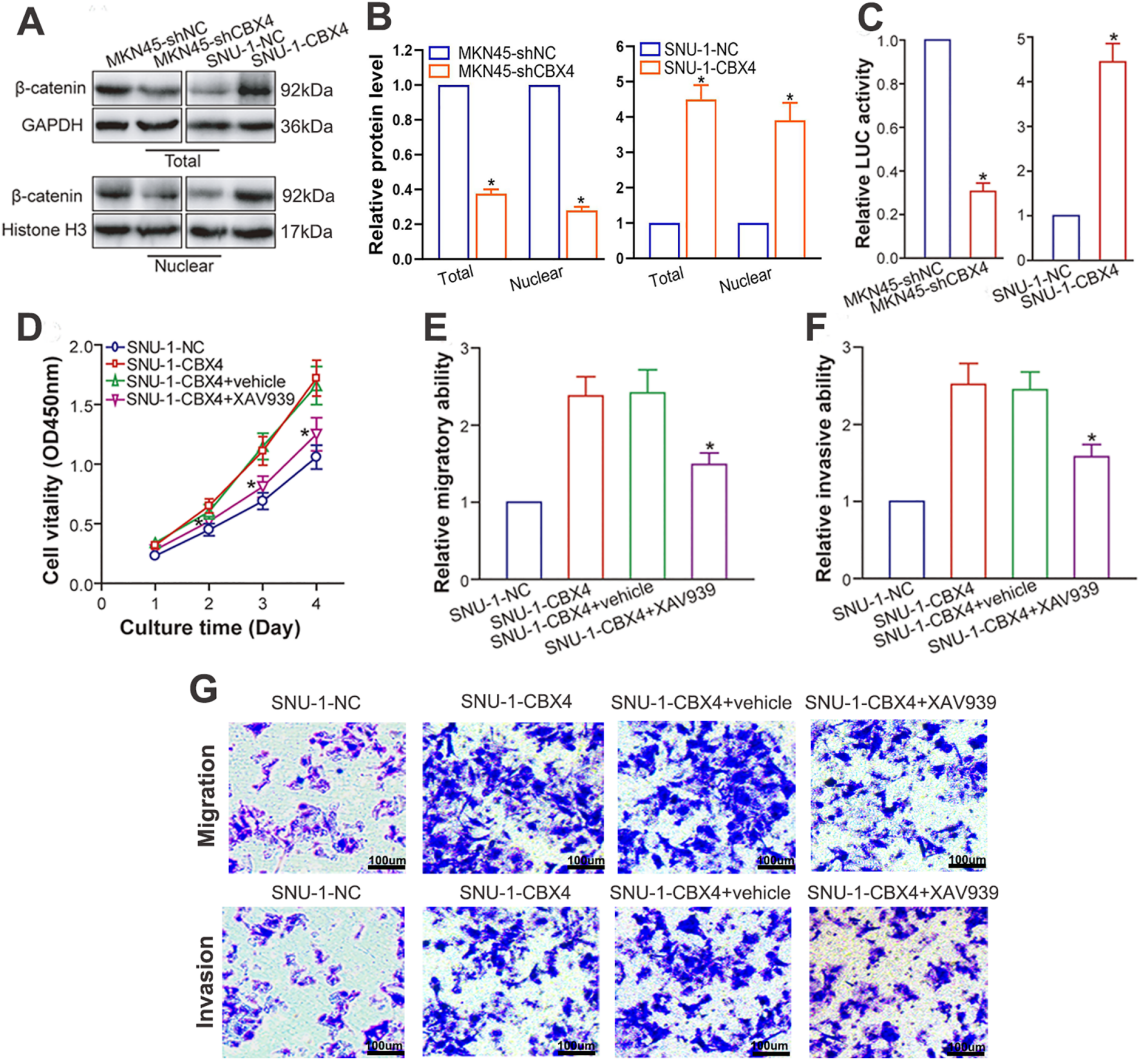
CBX4 facilitates GC progression via activation of β-catenin signaling. (**A**) Representative Western blot analysis of β-catenin protein levels in CBX4-modulated GC cell lines. (**B**) Quantification of total and nuclear β-catenin protein levels in CBX4-knockdown (MKN45-shCBX4 vs. MKN45-shNC) and CBX4-overexpressing (SNU-1-CBX4 vs. SNU-1-NC) GC cells. Values are normalized to respective controls (set to 1). *p < 0.05, Student’s *t*-test. (**C**) Relative firefly luciferase (β-catenin transcriptional) activity in CBX4-modulated GC cells. MKN45-shNC and SNU-1-NC served as controls for MKN45-shCBX4 and SNU-1-CBX4, respectively (set to 1). *p < 0.05, Student’s *t*-test. (**D**) CCK-8 assay results following Wnt/β-catenin inhibition. SNU-1-CBX4 cells treated with XAV939 showed significantly reduced cell viability compared to vehicle-treated or untreated SNU-1-CBX4 cells (*p < 0.05 at days 2, 3, and 4 post-culture), as assessed by ANOVA with LSD post hoc multiple comparisons. (**E**) Transwell migration assay results after Wnt/β-catenin inhibition. Relative migratory ability of SNU-1-CBX4, SNU-1-CBX4+vehicle, and SNU-1-CBX4+XAV939 cells, normalized to SNU-1-NC control (set to 1). SNU-1-CBX4+XAV939: *p < 0.05 vs. SNU-1-CBX4+vehicle or SNU-1-CBX4, ANOVA with LSD post hoc multiple comparisons. (**F**) Transwell invasion assay results following Wnt/β-catenin inhibition. Relative invasive ability of SNU-1-CBX4, SNU-1-CBX4+vehicle, and SNU-1-CBX4+XAV939 cells, normalized to SNU-1-NC control (set to 1). SNU-1-CBX4+XAV939: *p < 0.05 vs. SNU-1-CBX4+vehicle or SNU-1-CBX4, ANOVA with LSD post hoc multiple comparisons. (**G**) Representative images of transwell migration and invasion assays after Wnt/β-catenin inhibition

Wang et al. [23] documented a positive correlation between CBX4 and β-catenin at the protein level in human lung adenocarcinoma clinical samples, implying CBX4’s capacity to stimulate Wnt/β-catenin pathway activation. A wealth of studies have underscored the Wnt/β-catenin pathway’s significance in GC pathogenesis and progression [34,35]. To explore the molecular mechanism behind CBX4-induced GC progression and verify if CBX4 upregulation activates β-catenin signaling in GC cells, we examined β-catenin protein levels after CBX4 modulation in GC cells. WB analysis demonstrated that CBX4 knockdown markedly diminished total and nuclear β-catenin protein levels in MKN45 cells, whereas CBX4 overexpression elevated them in SNU-1 cells (*p* < 0.05; Fig. 5A and B). In line with this, luciferase reporter assays revealed that CBX4 depletion repressed, and CBX4 overexpression bolstered, β-catenin transcriptional activity (*p* < 0.05; Fig. 5C). Of critical importance, the oncogenic effects of CBX4 overexpression were robustly counteracted by the Wnt/β-catenin inhibitor XAV939 (*p* < 0.05; Figs. 5D–G). Collectively, these data substantiate that CBX4-mediated β-catenin signaling activation underlies the malignant biological behaviors of GC induced by CBX4.

## 6 Discussion

The dysregulation of CBX family proteins, which have been implicated in both promoting and suppressing tumorigenesis [36], highlights their complex roles in cancer biology. CBX4, a PcG-associated CBX protein with chromodomain and SIM domains, plays a crucial role in mediating biological processes such as gene silencing through PRC1 and SUMOylation via its E3 ligase activity. Recent studies have shown that CBX4 is closely associated with cancer, exhibiting both pro- and anti-tumor activities [25–27]. In GC, our study demonstrates that CBX4 upregulation is linked to malignant characteristics, including increased tumor size, lymph node metastasis, and higher TNM stage. Mechanistically, CBX4 promotes GC cell proliferation, metastatic potential, and β-catenin pathway activation.

As reported by Lin et al. [29] and Chen et al. [37], analysis of public databases reveals that GC tissues exhibit elevated mRNA and protein levels of CBX4 compared to normal tissues. To experimentally validate these findings, we evaluated CBX4 expression. IHC analysis of a GC TMA confirmed significant CBX4 upregulation in tumors compared to matched adjacent tissues. Consistently, WB analysis demonstrated that GC cells express higher levels of CBX4 protein compared to normal control cells. Notably, Yang et al. [38] recently reported that CBX4 mRNA is upregulated in GC through RT-qPCR analysis. Thus, our data, in conjunction with the data from Yang et al. [38], experimentally confirm the conclusions drawn by Lin et al. [29] and Chen et al. [37] from their database analysis. Further analysis revealed a correlation between high CBX4 protein expression and aggressive clinicopathological features, including large tumor size, lymph node metastasis positivity, and advanced TNM stage. This parallels earlier associations between CBX4 mRNA levels and disease stage or lymph node status [29,37]. In terms of prognosis, Lin et al. [29] reported significant correlations between high CBX4 mRNA levels and reduced overall survival (OS), progression-free survival (PFS), and post-progression survival (PPS). In contrast, Chen et al. [37] observed non-significant trends for OS and PFS. Our independent Kaplan-Meier analysis (n = 875 GC patients) corroborated Lin et al.’s findings regarding OS. One possible explanation for the discrepancies in these prognostic correlation findings is the difference in the selected databases and analysis methods. Nevertheless, with the follow-up survival data from our enrolled 114 GC patients, the relationship between CBX4 protein levels and GC prognosis will be further elucidated, providing a foundation for understanding the correlation between CBX4 and GC prognosis.

Dysregulated cell growth and dissemination are core characteristics of cancer [39–41]. Epigenetic modifying molecules can drive oncogenesis and therapeutic resistance through diverse pathological pathways [42–44]. Accumulating evidence indicates that CBX4 promotes proliferation, growth, and metastasis in various cancers, including HCC [18,20], breast cancer [21,45], lung cancer [22,23], osteosarcoma [24,25], and kidney cancer [26]. Additionally, CBX4 plays a key role in the pro-oncogenic activities of several oncogenic RNAs, such as LINC00265 [38], FOXP4-AS1 [46], circ_PVT1 [47], circRNA_100876 [48], and circ_0008039 [49]. Conversely, tumor suppressor miRNAs like miR-129-5p [50] and miR-497-5p [51] exert their tumor-suppressive effects through CBX4 inhibition. Thus, CBX4 significantly contributes to malignant phenotypes. To explore the functional and clinical significance of CBX4 in GC, we developed transgenic GC cell models with modulated CBX4 expression. Comprehensive functional analyses, including *in vitro* assays and *in vivo* xenograft models, showed that CBX4 knockdown inhibits GC cell proliferation, migration, invasion, and tumor growth capacity. In contrast, CBX4 overexpression enhances these oncogenic properties. Our experimental data therefore provide mechanistic insights into the clinical association between high CBX4 expression in GC tissues and adverse clinicopathological features, confirming CBX4 as a key pro-oncogenic factor in GC progression.

The Wnt/β-catenin signaling pathway plays a pivotal role in orchestrating stemness, proliferation, differentiation, motility, and development [52,53], and its dysregulated activation is implicated in various pathologies, particularly cancer [54,55]. In GC, this pathway is critical for driving oncogenesis, tumor growth, invasion, metastasis, immune evasion, and treatment resistance [34,35], highlighting the imperative to elucidate its regulatory mechanisms. Previous studies have implicated CBX4 in Wnt/β-catenin activation in lung adenocarcinoma (showing a positive correlation with β-catenin levels) [23] and laryngeal cancer (via the circ_PVT1/miR-21-5p/CBX4 axis) [53]. To investigate CBX4’s role in GC pathogenesis, we examined its interaction with β-catenin. Our experiments revealed that CBX4 depletion significantly reduced total and nuclear β-catenin protein levels and suppressed β-catenin-dependent transcriptional activity. In contrast, CBX4 overexpression elevated these parameters. Notably, treatment with the Wnt/β-catenin inhibitor XAV939 attenuated the oncogenic effects induced by CBX4 overexpression. Collectively, our findings demonstrate that CBX4 predominantly enhances GC malignancy by amplifying β-catenin signaling.

Reports indicate that lncRNA, circRNA, and miRNA are involved in regulating CBX4 expression. In various tumors, multiple miRNAs targeting CBX4 have been identified, such as miR-21-5p [47], miR-129-5p [50], miR-136-5p [46,48], miR-144-3p [38], miR-497-5p [51], and miR-515-5p [49]. The downregulation of these miRNAs, typically mediated by lncRNA and circRNA via a competing endogenous RNA (ceRNA) mechanism, results in CBX4 upregulation in tumor cells. For example, circ_PVT1 upregulation can increase CBX4 expression by targeting and repressing miR-21-5p in laryngeal cancer [47]. FOXP4-AS1 [46] and circRNA_100876 [48] can competitively bind to miR-136-5p, which targets CBX4 in cervical and bladder cancers, respectively, leading to CBX4 upregulation. Additionally, circ_0008039 upregulates CBX4 by competitively binding to miR-515-5p in breast cancer [49]. In GC, LINC00265 can act as a ceRNA for miR-144-3p to upregulate CBX4 [38]. These reports suggest that non-coding RNA-mediated post-transcriptional regulation is a significant mechanism for upregulating CBX4 expression in tumor cells, which may better explain why both CBX4 mRNA and protein levels are elevated in GC. Although the mechanism of LINC00265/miR-144-3p/CBX4 axis-mediated CBX4 upregulation in GC has been identified [38], it remains to be further studied whether other non-coding RNAs regulate CBX4 in GC. More recently, the carboxyl terminus of HSC70-interacting protein (CHIP) was found to be an upstream negative regulator of CBX4 in osteosarcoma [25]. Inactivation of the CK1α/CHIP axis induces CBX4 expression in osteosarcoma cells, whereas restoring its activity mediates CBX4 ubiquitination and degradation, thereby downregulating CBX4 protein levels through post-translational regulation. It is highly interesting to investigate whether CK1α/CHIP axis-mediated post-translational regulation of CBX4 occurs in GC, a question requiring further exploration in our future work. If such a post-translational regulatory mechanism exists, we propose a feedforward model: during Wnt/β-catenin signaling activation, inactivation of the CK1α/β-TrCP axis activates β-catenin signaling. Concurrently, inactivation of the CK1α/CHIP axis upregulates CBX4, which promotes β-catenin signaling activation. The upregulated CBX4 may serve as a mediator to amplify β-catenin signaling activation, thereby enhancing our understanding of CBX4’s role in GC cells. In summary, studying the molecular regulatory mechanisms underlying CBX4 upregulation in GC holds great biological and clinical significance.

Although we demonstrated that CBX4 activates β-catenin signaling and its transcriptional activity, we did not identify or validate the specific downstream target genes (e.g., *c-MYC*, *CCND1*, *AXIN2*) responsible for mediating the oncogenic effects of CBX4 in gastric cancer. Confirming the altered expression of these classic Wnt/β-catenin targets following CBX4 modulation would have strengthened the mechanistic link between CBX4 and the pathway’s transcriptional output.

Collectively, our study identifies CBX4 as an upregulated oncogenic driver in gastric cancer. The CBX4-mediated enhancement of β-catenin signaling is central to gastric cancer progression. Consequently, CBX4 is not only a novel prognostic indicator but also a promising therapeutic target. Thus, disrupting the CBX4/β-catenin signaling axis pharmacologically may offer an effective strategy for gastric cancer treatment.

## Data Availability

The data that support the findings of this study are available from the corresponding authors upon reasonable request.
